# Supplementary material for: A fault-intrusion-tolerant system and deadline-aware algorithm for scheduling scientific workflow in the cloud
Source: PeerJ Comput Sci. 2021 Nov 2;7:e747. doi: 10.7717/peerj-cs.747 (PMC8576554; doi:10.7717/peerj-cs.747)
Supplement: Supplemental Information 6 [file peerj-cs-07-747-s006.zip › Myproject1.1/docs/org/cloudbus/cloudsim/network/datacenter/package-tree.html]

org.cloudbus.cloudsim.network.datacenter Class Hierarchy


JavaScript is disabled on your browser.


- Overview
- Package
- Class
- Use
- Tree
- Deprecated
- Index
- Help

- Prev
- Next

- Frames
- No Frames

- All Classes

# Hierarchy For Package org.cloudbus.cloudsim.network.datacenter

Package Hierarchies:

- All Packages

## Class Hierarchy

- java.lang.Object
  - org.cloudbus.cloudsim.network.datacenter.AppCloudlet
    - org.cloudbus.cloudsim.network.datacenter.WorkflowApp
  - org.cloudbus.cloudsim.Cloudlet
    - org.cloudbus.cloudsim.network.datacenter.NetworkCloudlet (implements java.lang.Comparable<T>)
  - org.cloudbus.cloudsim.CloudletScheduler
    - org.cloudbus.cloudsim.network.datacenter.NetworkCloudletSpaceSharedScheduler
  - org.cloudbus.cloudsim.Host
    - org.cloudbus.cloudsim.network.datacenter.NetworkHost
  - org.cloudbus.cloudsim.network.datacenter.HostPacket
  - org.cloudbus.cloudsim.network.datacenter.NetworkConstants
  - org.cloudbus.cloudsim.network.datacenter.NetworkPacket
  - org.cloudbus.cloudsim.core.SimEntity (implements java.lang.Cloneable)
    - org.cloudbus.cloudsim.Datacenter
      - org.cloudbus.cloudsim.network.datacenter.NetworkDatacenter
    - org.cloudbus.cloudsim.network.datacenter.NetDatacenterBroker
    - org.cloudbus.cloudsim.network.datacenter.Switch
      - org.cloudbus.cloudsim.network.datacenter.AggregateSwitch
      - org.cloudbus.cloudsim.network.datacenter.EdgeSwitch
      - org.cloudbus.cloudsim.network.datacenter.RootSwitch
  - org.cloudbus.cloudsim.network.datacenter.TaskStage
  - org.cloudbus.cloudsim.Vm
    - org.cloudbus.cloudsim.network.datacenter.NetworkVm (implements java.lang.Comparable<T>)
  - org.cloudbus.cloudsim.VmAllocationPolicy
    - org.cloudbus.cloudsim.network.datacenter.NetworkVmAllocationPolicy

- Overview
- Package
- Class
- Use
- Tree
- Deprecated
- Index
- Help

- Prev
- Next

- Frames
- No Frames

- All Classes
